# DNase Sda1 Allows Invasive M1T1 Group A *Streptococcus* to Prevent TLR9-Dependent Recognition

**DOI:** 10.1371/journal.ppat.1002736

**Published:** 2012-06-14

**Authors:** Satoshi Uchiyama, Federica Andreoni, Reto A. Schuepbach, Victor Nizet, Annelies S. Zinkernagel

**Affiliations:** 1 Division of Infectious Diseases and Hospital Epidemiology, University Hospital Zurich, University of Zurich, Zurich, Switzerland; 2 Division of Surgical Intensive Care, University Hospital Zurich, University of Zurich, Zurich, Switzerland; 3 Department of Pediatrics, Division of Pharmacology & Drug Discovery and Skaggs School of Pharmacy & Pharmaceutical Sciences, University of California, San Diego, La Jolla, California, United States of America; National Institute of Allergy and Infectious Diseases, National Institutes of Health, United States of America

## Abstract

Group A *Streptococcus* (GAS) has developed a broad arsenal of virulence factors that serve to circumvent host defense mechanisms. The virulence factor DNase Sda1 of the hyperinvasive M1T1 GAS clone degrades DNA-based neutrophil extracellular traps allowing GAS to escape extracellular killing. TLR9 is activated by unmethylated CpG-rich bacterial DNA and enhances innate immune resistance. We hypothesized that Sda1 degradation of bacterial DNA could alter TLR9-mediated recognition of GAS by host innate immune cells. We tested this hypothesis using a dual approach: loss and gain of function of DNase in isogenic GAS strains and presence and absence of TLR9 in the host. Either DNA degradation by Sda1 or host deficiency of TLR9 prevented GAS induced IFN-α and TNF-α secretion from murine macrophages and contributed to bacterial survival. Similarly, in a murine necrotizing fasciitis model, IFN-α and TNF-α levels were significantly decreased in wild type mice infected with GAS expressing Sda1, whereas no such Sda1-dependent effect was seen in a TLR9-deficient background. Thus GAS Sda1 suppressed both the TLR9-mediated innate immune response and macrophage bactericidal activity. Our results demonstrate a novel mechanism of bacterial innate immune evasion based on autodegradation of CpG-rich DNA by a bacterial DNase.

## Author Summary

Group A *Streptococcus* (GAS) ranks among the top ten human pathogens causing fatal disease. GAS possesses an arsenal of virulence factors that circumvent the primary mammalian defence strategies, the innate immune system. Toll-like receptors (TLRs), allow the host to detect pathogens by recognizing structures or patterns abundant in pathogens but lacking in the mammalian host, including unmethylated CpG-rich bacterial DNA recognized by TLR9. Here we show that GAS DNA but not host DNA triggers TNF-α and interferon type 1 cytokine secretion by monocytic cells, and that this secretion is dependent on the presence of functional TLR9. The highly virulent M1T1 GAS clone expresses the virulence factor DNase Sda1. Sda1-mediated bacterial DNA degradation was shown to prevent TLR9-dependent cytokine release in monocytes, which then fail to effectively phagocytose and kill bacteria. In a mouse necrotizing fasciitis model, the streptococcal DNase Sda1 suppressed TLR9-dependent INF-α and TNF-α induction. Inhibition of TLR9 recognition by a bacterial DNase thus illustrates a novel mechanism of microbial innate immune evasion.

## Introduction

The Gram-positive bacterium Group A *Streptococcus* (GAS) is a leading human pathogen, annually causing over 700 million cases of superficial infections such as pharyngitis or pyoderma, and more than 650,000 cases of invasive infections, including the potentially lethal conditions of necrotizing fasciitis (NF) and streptococcal toxic shock syndrome (STSS) [1]. Increased reports of severe GAS disease in recent decades have been in large part attributable to the emergence of a globally disseminated clone of the M1T1 serotype [2]. M1T1 strains are the most common cause of GAS pharyngitis and are strongly overrepresented in severe cases such as NF and STSS [3]. The ability of invasive GAS to produce life-threatening infections even in previously healthy individuals reflects a diverse array of virulence factors that together allow the bacterium to invade host cellular barriers and resist innate immune clearance [2], [4]. One important distinguishing feature of the globally-disseminated M1T1 GAS clone compared to less pathogenic GAS strains is the acquisition of a prophage encoding a potent secreted DNase, Sda1 [5]. Sda1 activity has been shown to promote GAS escape from phagocytic killing with DNA-based neutrophil extracellular traps (NETs) [6], [7], [8], frameworks of DNA containing antimicrobial peptides, histones and proteases that are generated by neutrophils to capture and eliminate bacteria at tissue foci of infection [9].

To control an infection quickly and to prevent disease progression, timely and accurate recognition of bacteria by the host innate immune system is crucial. Pattern recognition receptors (PRRs) such as Toll like receptors (TLRs) recognize conserved molecular patterns from pathogens. The Toll-like receptor 9 (TLR9) is located intracellularly and recognizes unmethylated CpG-rich DNA motifs commonly present in microorganisms but absent in the host genome [10]. Due to its intracellular localization, TLR9 was first appreciated to respond to intracellular pathogens such as *Listeria monocytogenes* and *Legionella pneumophila* [11], [12]. However, TLR9 has recently been shown to enhance resistance against the common Gram-positive bacteria *Streptococcus pneumoniae* [13] and GAS [14]. Following this lead, we hypothesized that the presence of the potent DNase Sda1 in the hyperinvasive M1T1 GAS clone could modify its own unmethylated extracellular CpG-rich DNA fragments and alter TLR9-mediated recognition by host innate immune cells. Combining studies with M1T1 GAS and an isogenic strain with loss of Sda1 expression with macrophages derived from WT and TLR-9 deficient mice, we demonstrate a novel mechanism of bacterial innate immune evasion based on autodegradation of a key pattern-recognition molecule.

## Results/Discussion

### GAS genomic DNA results in IFN-α and TNF-α secretion by murine macrophages in a dose and time dependent manner

**Figure 1:**
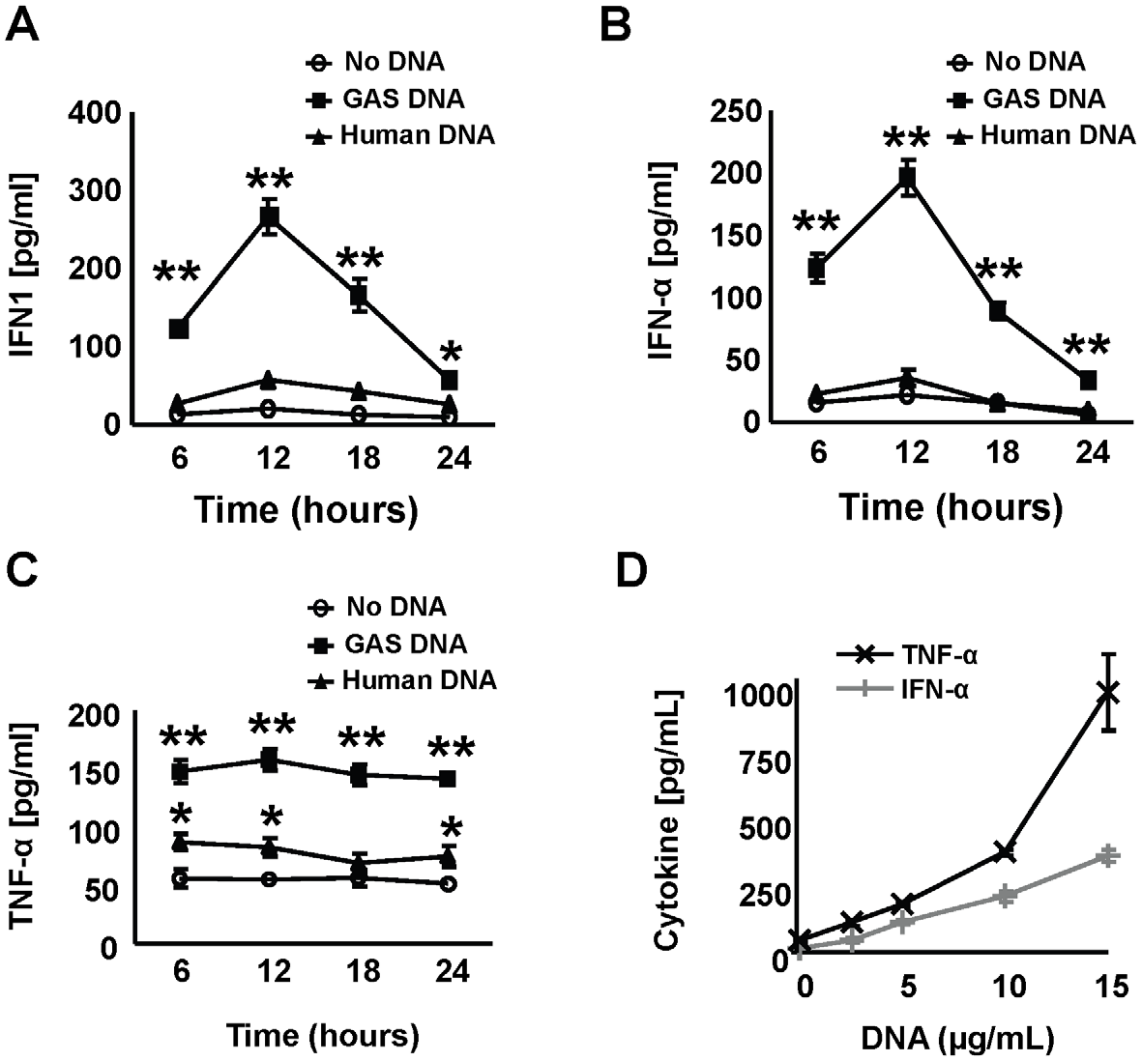
Stimulation of murine macrophages by human and GAS DNA. (**A–C**) Bone marrow-derived mice macrophages (BMDMs) were stimulated with GAS and human genomic DNA (both 5 µg/ml) and secretion of IFN1, IFN-α and TNF-α in the supernatants measured. (**D**) Dose-dependency of GAS DNA-mediated stimulation (12 hours) of IFN-α and TNF-α secretion. Data were pooled from 3 experiments done in triplicates and presented as mean ± SEM. * *P*<0.05 ** *P*<0.01.

DNA was purified from GAS strain 5448, representative of the globally disseminated hyperinvasive M1T1 clone, and incubated with murine bone marrow-derived macrophages (BMDMs). Cytokine release into the medium was used as readout for BMDM activation. GAS DNA induced time-dependent release of interferon type 1 (IFN-1), and specifically interferon-α (IFN-α), from the macrophages (**Fig. 1A, B**), peaking at 12 h of exposure. GAS DNA also induced BMDM TNF-α secretion, with maximal levels already detected after 6 h of incubation and remaining elevated for at least 24 h (**Fig. 1C**). Induction of IFN-α and TNF-α release by GAS DNA was also dose-dependent (**Fig. 1D**). In contrast, human DNA did not induce IFN-1 or TNF-α secretion from BMDMs (**Fig. 1A–C**). We did not detect specific induction of the cytokines IL-6, IL-1β, IL-10 or MIP-2 from BMDMs exposed to GAS DNA (data not shown).

Unmethylated CpG-rich DNA motifs have previously been reported to induce macrophage secretion of proinflammatory cytokines including TNF-α and IFN-1 [10], [15]. Further studies have shown that macrophages exposed to live GAS, DNA isolated from GAS or antibiotic-killed group B *Streptococcus* release IFN-β and TNF-α [16], [17]. However, bacterial DNA has not previously been shown to stimulate IFN-α secretion, a finding relevant to innate immune defense since IFN-α is known to provide protection against Gram-positive bacterial infections [18].

### Stimulation and secretion of IFN-α and TNF-α in murine macrophages is TLR9 dependent

**Figure 2:**
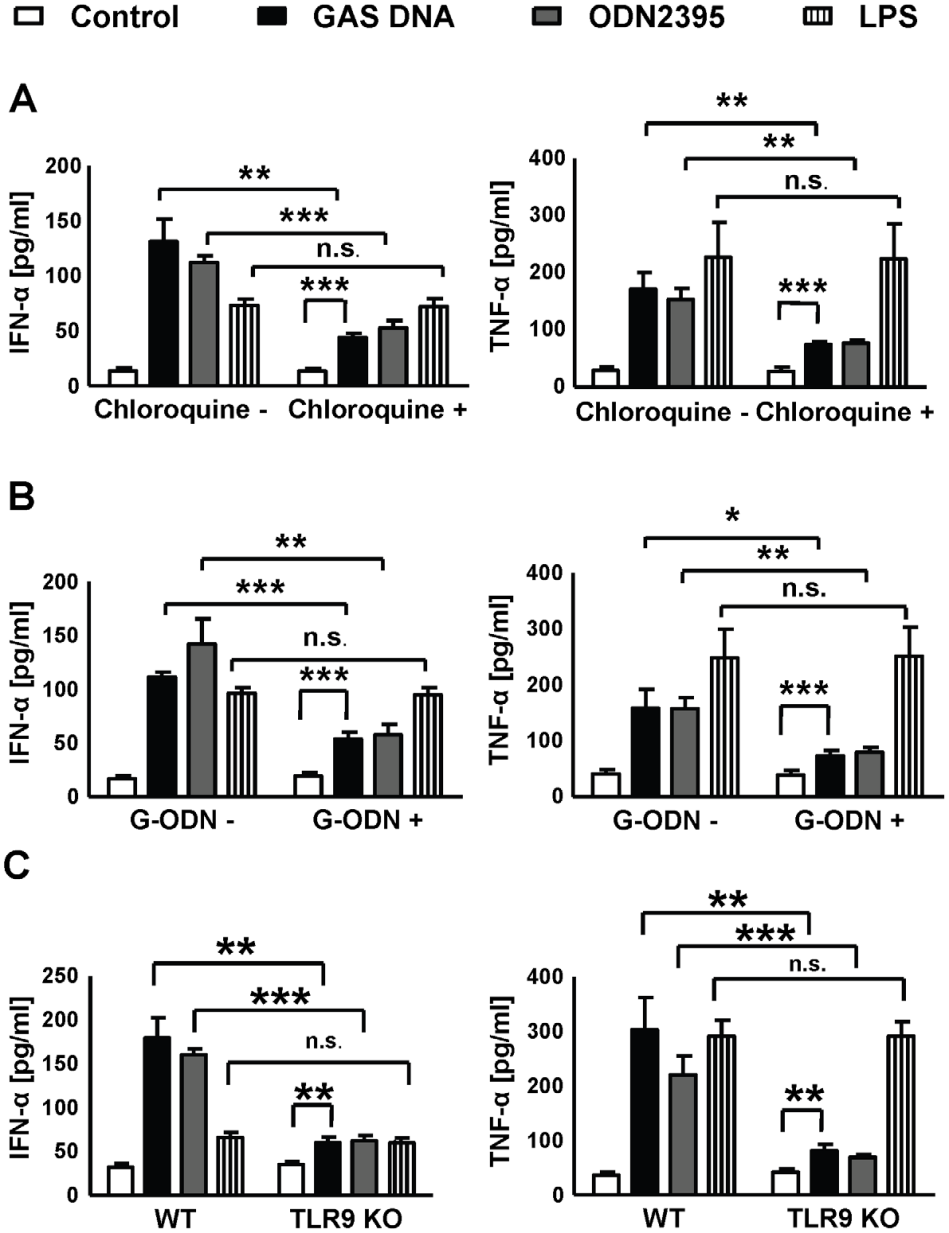
GAS DNA stimulation of murine macrophages is TLR9-dependent. (**A, B and C**) To test for TLR9-dependency of GAS DNA-mediated BMDMs stimulation, the known inhibitors of the TLR9 pathway cloroquine (100 µM) and G-ODN (10 µM) as well as the stimulator ODN2395 (1 µM) were used together with BMDMs derived from wild type and TLR9-deficient mice. LPS (10 ng/ml) was used as a positive control in experiments with perturbed TLR9 signalling. The addition of chloroquine or G-ODN and the use of TLR9-deficient BMDMs significantly reduced the GAS DNA and ODN2395-mediated production of IFN-α and TNF-α while stimulation mediated by LPS was unaffected. Data were pooled from 3 experiments done in triplicate and presented as mean ± SEM. *P<0.05 ** *P*<0.01 *** *P*<0.001.

The experiments above showed that GAS DNA, containing unmethylated CpG motifs, but not human DNA, induced IFN-α and TNF-α release by BMDM. Since IFN type 1 secretion is partially mediated by TLR9 [19], we tested whether cytokine release in murine BMDMs expressing TLR9 [20], [21], [22], occurred in a TLR9-dependent manner. Chloroquine blocks endosomal acidification and is a known inhibitor of TLR9 [23], [24]. We observed a significant decrease of IFN-α and TNF-α secretion in response to GAS DNA and to the TLR9 agonist ODN2395 in BMDMs pretreated with chloroquine, whereas TLR4-mediated responses to LPS were unaffected (**Fig. 2A**). Similar results were obtained with the synthetic TLR9 antagonist G-ODN (**Fig. 2B**). To further corroborate the TLR9 dependency, experiments were repeated with BMDMs extracted from TLR9-deficient mice. Stimulation using the TLR9 agonist ODN2395 induced BMDM secretion of IFN-α and TNF-α only in the presence of a functional TLR9 pathway, whereas responses to LPS were not influenced (**Fig. 2C**). Similarly, after stimulation with GAS DNA, a significantly lower release of IFN-α and TNF-α was observed from TLR9-deficient compared to WT BMDMs (**Fig. 2C**). The stimulation of IFN-α secretion from TLR9-deficient BMDMs, albeit at a reduced level, is most likely explained by a ubiquitous interferon response to immunostimmulatory nucleic acids, mediated by cytosolic DNA sensors amongst others [25]. Similarly, recent work shows that IFN-β is secreted after challenge of TLR9-deficient macrophages with live GAS or GAS DNA complexed with RNA [16].

### M1T1 GAS DNase Sda1 diminishes TLR9-dependent IFN-α and TNF-α secretion by macrophages

**Figure 3:**
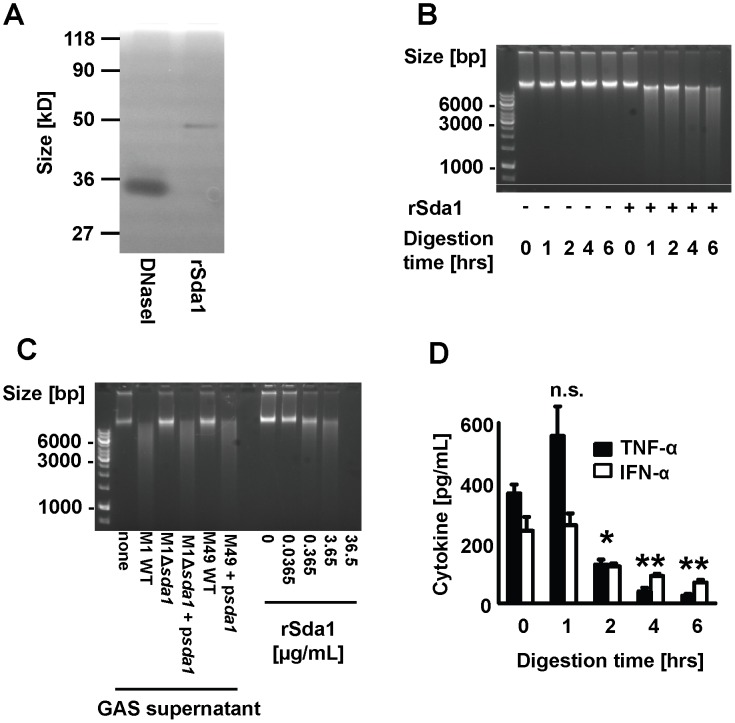
The recombinant GAS DNase Sda1 degrades genomic DNA. (**A**) Zymogram and (**B** and **C**) agarose gel electrophoresis were used to visualize degradation of DNA by rSda1. (**B**) The size of the GAS DNA treated with rSda1 for 0 to 6 hours was analyzed by agarose gel electrophoresis. (**C**) Supernatants of the GAS strains used in the assays were co-incubated with DNA. DNA degradation was assessed by agarose gel electrophoresis: the degradation efficiency displayed by GAS supernatants was directly compared to the degradation efficiency of different concentrations of rSda1. (**D**) GAS DNA was pre-incubated with rSda1 for 0 to 6 hours before the reaction mixture was tested for its capacity to induce cytokine secretion in BMDMs. The data were pooled from 3 experiments done in triplicates and presented as mean ± SEM., *P<0.05, **P<0.01 as compared to the 0 hour time point.

**Figure 4:**
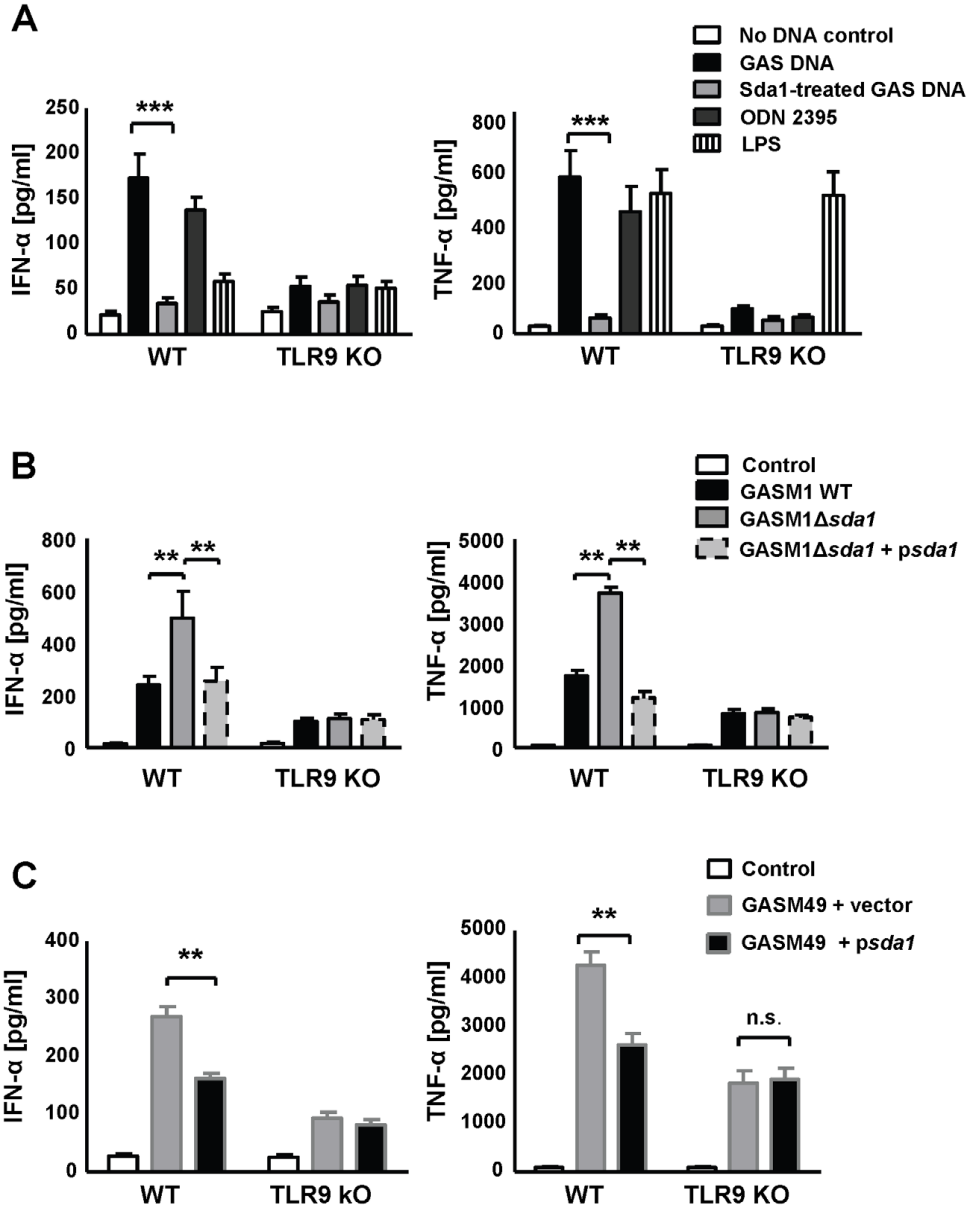
The GAS DNase Sda1 interferes with TLR9 activation. (**A**) IFN-α and TNF-α secretion by BMDMs after GAS DNA challenge for 12 hours was almost abolished after adding recombinant Sda1 to GAS DNA. (**B** and **C**) Stimulation of BMDMs by GAS strains expressing Sda1 (GASM1 WT and GASM49 pD*csda1*) for 12 hours secreted significantly less IFN-α and TNF-α compared to matching strains lacking Sda1 (GASM1 Δ*sda1* and GASM49 WT pD*cerm*). Data were pooled from 3 experiments done in triplicates and presented as mean ± SEM. ** *P*<0.01 *** *P*<0.001.

An important characteristic of the hypervirulent globally disseminated M1T1 clone of GAS is the presence of a prophage-encoded secreted DNase, *sda1* [5]. Sda1 has been shown to promote M1T1 GAS virulence via degradation of NETs, allowing the bacteria to escape neutrophil killing and the tissue focus of infection, thus facilitating systemic spread of the pathogen [2], [6], [7]. Functional TLR9 is important in defense against GAS infection [14] and the DNA size required for optimal stimulation varies among host cells. Whereas B-cells are stimulated by small DNA fragments [26], macrophages show enhanced uptake and subsequent responses with increasing DNA length [26]. Having observed efficient BMDM activation by crude GAS DNA (above) we hypothesized that degradation by Sda1 could reduce stimulation of macrophage and thus be an additional immune evasion function of Sda1. To test this, we engineered recombinant GAS Sda1 (rSda1) in *E. coli*. Purification yielded a 45 kD recombinant protein which showed DNase activity at the expected size when analyzed by zymography (**Fig. 3A**). Recombinant Sda1 degraded GAS DNA in a time and concentration dependent manner (**Fig. 3B–C**). Recombinant Sda1 at around 4 µg/mL was similarly efficient in degrading DNA as the natively or overexpressed Sda1 in GAS supernatants (**Fig. 3C**). Degradation of GAS DNA by Sda1 abolished induction of TNF-α and IFN-α in BMDM's (**Fig. 3D**). DNase Sda1 on its own did not influence cytokine secretion (**Fig. S2**). Similarly DNase Sda1 treatment of GAS DNA did not affect the residual level of IFN-α and TNF-α induction when TLR9-deficient BMDMs were studied (**Fig. 4A**). We speculate that the decreased TLR9-dependent cytokine responses to Sda1-treated GAS DNA was mainly due to decreased average DNA size (**Fig. 3B**), which has also been shown by others to be crucial for cellular uptake of DNA and subsequent TLR9 stimulation [26]. In addition direct elimination of CpG motifs by the efficient enzymatic action of the bacterial DNase [10] could potentially further contribute to the differences observed. Since it has been reported that Sda1 can degrade RNA [27] and recent work shows that IFN-β is secreted by macrophages after challenge of GAS DNA complexed with RNA [16] we investigated the action of Sda1 against RNA, in addition to DNA, wondering if this could be a two-pronged approach to promote GAS infection. RNA co-incubated with our rSda1 showed no degradation when visualized by agarose gel electrophoresis (**Fig. S1**), suggesting that Sda1 possesses negligible or minor RNA-degrading activity under our assay conditions. To study the influence of Sda1 on TLR9-mediated macrophage responses to live GAS infection, BMDMs were challenged with the wild type M1TI GAS parent strain M1 5448 (M1WT), the isogenic GAS DNase *sda1* knockout (M1Δ*sda1*) and the *sda1* complemented strain (M1Δ*sda1*pDc*sda1*) using the pDc*sda1* plasmid [6]. A significant increase in IFN-α and TNF-α secretion was observed from WT BMDMs challenged with the M1Δ*sda1* mutant strain compared to the parent and complemented strains (**Fig. 4B**). The observed Sda1-dependent reduction of cytokine responses to GAS was diminished in TLR9-deficient macrophages (**Fig. 4B**). Similarly, heterologous expression of *sda1* in a less virulent M49 GAS strain diminished BMDM IFN-α and TNF-α secretion in a TLR9-dependent manner (**Fig. 4C**). Our paired loss- and gain-of-function analyses indicate that Sda1 is both necessary and sufficient to promote GAS avoidance of TLR9-dependent macrophage recognition [10].

TLR9 is activated by CpG-rich DNA motifs present in most bacteria. Evolution of reduced CpG content (CpG suppression) has been described in other microorganisms including nonpathogenic viruses, *Plasmodium falciparum* and *Entamoeba histolytica* [28]. In contrast GAS DNA possesses a high CpG content. By acquiring a potent secreted DNA-degrading enzyme, GAS has come across an alternative means to circumvent TLR9 activation in the host innate immune response.

### GAS DNase Sda1 promotes resistance to macrophage intracellular killing

**Figure 5:**
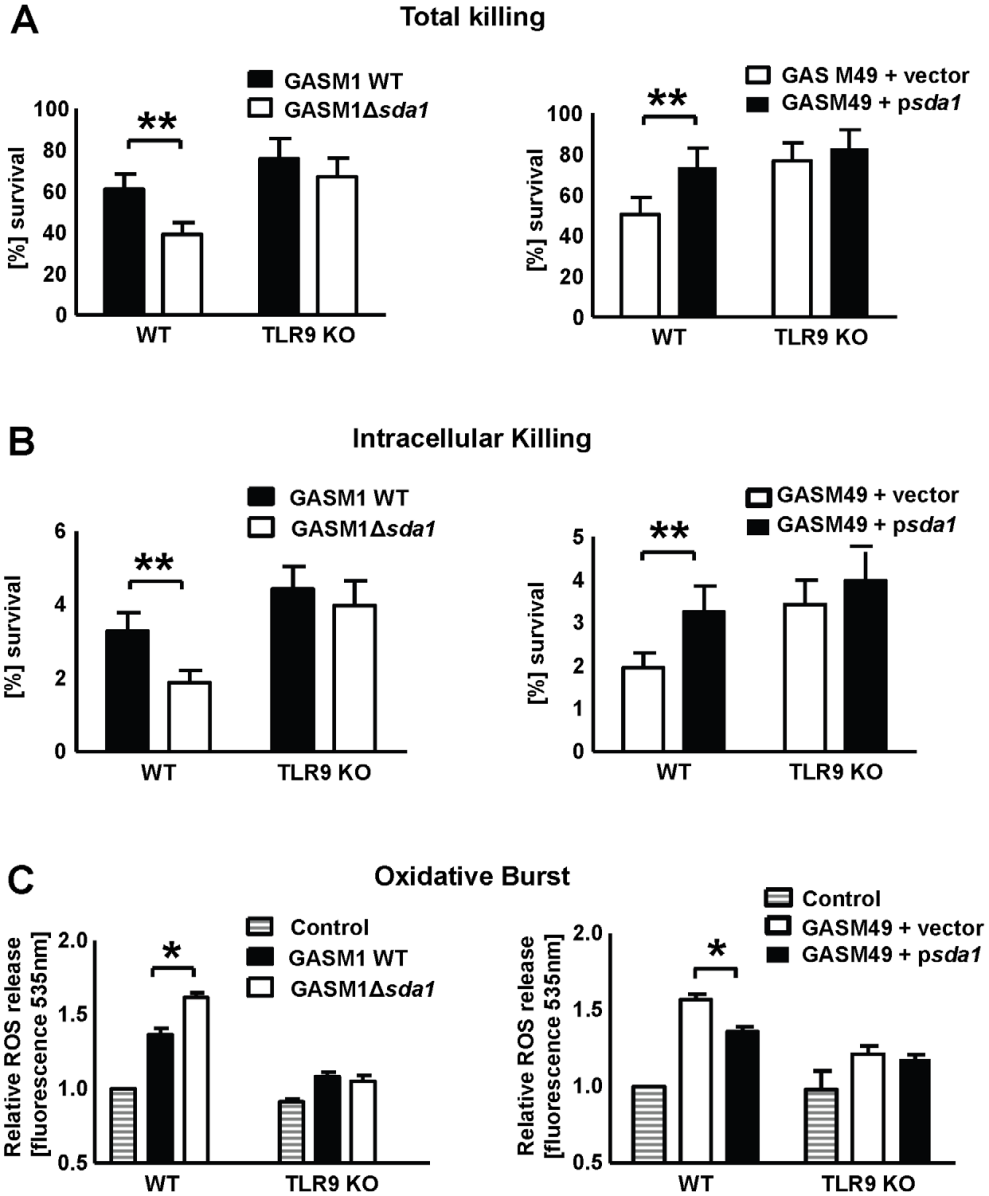
GAS DNase Sda1 promotes resistance to macrophage killing. (**A** and **B**) BMDMs were inoculated at an MOI = 1 with GAS M1 and M49 strains expressing or lacking Sda1. A significantly higher survival rate, for both total (**A**) and in intracellular killing (**B**), was seen for GAS strains expressing Sda1. Differences in killing could not be observed in BMDMs derived from TLR9-deficient mice. (**C**) Oxidative burst of WT and TLR9-deficient BMDMs was measured as fluorescent units at 535 nm after 30 min stimulation with GAS M1 or GAS M49 strain either expressing or lacking Sda1. The oxidative burst fluorescent units of the non-stimulated control macrophages were set at 1. The presence of Sda1 decreased oxidative burst in WT BMDMs while no effect was detected in TLR9-deficient BMDMs. Data used in Fig. 5A and B were pooled from 3 experiments done in triplicate. Data shown in Fig. 5C is one out of three representative experiments each done in triplicate and presented as mean ± SEM (A and B) ± SD (C). * *P*<0.05 ** *P*<0.01.

To date, DNase Sda1 has been appreciated to promote M1T1 GAS resistance to neutrophil extracellular killing due to its capacity to digest NETs [6], [7], [8]. Since we here identified a capacity of Sda1 to diminish TLR9-mediated macrophage responses, we hypothesized that the DNase activity could blunt the innate immune killing capacity of macrophages to kill GAS. Mice depleted of macrophages or treated with inhibitors of macrophage phagocytosis cannot clear GAS infections even at relatively low challenge doses [29], demonstrating the essential first line defense function of these immune cells against the pathogen. WT and TLR9-deficient BMDMs were challenged with live M1 or M49 GAS either expressing or not DNase and total, intra and extracellular bacterial killing was quantified. GAS strains expressing Sda1 survived significantly better in both the total and intracellular killing assays compared to the strains in which Sda1 was not expressed (**Fig. 5A, B** **and S3**). The Sda1-mediated survival advantages for GAS were much more pronounced in WT compared to TLR9-deficient BMDMs.

Sda1-mediated resistance to total macrophage killing could be caused by interference with DNA-based extracellular traps, which, we have recently observed, are generated by macrophages [30] though to a much lesser extent than observed in neutrophils or mast cells exposed to GAS. To investigate if the bacterial DNase Sda1 interferes with extracellular killing by macrophages we pretreated the macrophages with cytochalasin D to inhibit phagocytosis. No difference between GAS WT and the isogenic GAS DNase *sda1* knockout strains were observed for the extracellular killing indicating that the Sda1-dependent survival advantage seen *in vitro* is indeed mainly intracellular (**Fig. S4**). To further explore the Sda1-dependent survival advantage of GAS intracellularly following phagocytotic uptake into the macrophages, we measured oxidative burst activity, which has been reported to be a TLR9-induced mediator of intracellular killing in murine macrophages [14]. Oxidative burst activity was measured in WT and TLR9-deficient BMDMs after infection with the isogenic pairs of GAS strains either expressing or lacking DNase Sda1. BMDMs infected with GAS strains possessing Sda1 displayed a significantly reduced oxidative burst response compared to BMDMs infected with GAS strains lacking Sda1 (**Fig. 5C**). We propose that reduced oxidative burst is an additional mechanism by which Sda1 can contribute to M1T1 GAS resistance to macrophage killing. In order to test if blocking IFN-α and TNF-α can prevent phagocytic killing mediated by GASΔ*sda1* we repeated the BMDM killing assays with WT BMDM challenged with WT GAS M1 and GASΔ*sda1* bacteria after having pre-incubated the BMDM with either the neutralizing antibodies against TNF-α or IFN-α or their respective controls. Addition of neutralizing IFN-α antibodies increased the survival of GAS and GASΔ*sda1* when challenged with BMDMs from WT mice. The effect of neutralizing TNF-α antibodies was smaller and not statistically significant upon challenge with WT GAS and GASΔ*sda1* mutant bacteria (**Fig. S7**). These results suggest that certain cytokines may themselves contribute to enhance the phagocytic killing of bacteria.

Viability of the BMDMs was >90% after 4 and 12 h of stimulation with the bacteria at MOI of 1. No significant differences were observed in survival of WT or TLR9 deficient BMDMs stimulated with either GAS WT or the GASΔ*sda1* mutant (**Fig. S6**).

All GAS strains are known to express DNase activity, and some strains produce up to 4 different DNases. As first described by Wannemaker [27], [31] these proteins were designated DNase A, B, C and D. However the role of theses DNases remained unclear until 50 years later when it was shown that GAS DNase activity, particularly that of GAS DNase D (now known as Sda1) was important for virulence [6], [8]. By creating knockout mutants of the three DNases present in the GAS MGAS5005 strain [8], Sumby et al. determined that Sda1 was the most active. Sda1 very efficiently degraded DNA *in vitro*. In murine skin infection models, engineered GAS strains expressing Sda1 alone were found to be as virulent as wild-type GAS, supporting the conclusion that Sda1 but no other DNases mediate virulence *in vivo*. The strong activity of Sda1 compared to the weaker DNA-degrading activity of the other DNases found in GAS, may help to explain the pronounced phenotype we have observed in enhancing TLR9-mediated clearance when only Sda1, and no other DNases, is knocked out. However our data may underestimate the full collective potential of GAS DNases in TLR9-mediated innate immune evasion.

### The GAS DNase Sda1 diminishes local secretion of IFN-α and TNF-α in a mouse necrotizing fasciitis skin infection model

**Figure 6:**
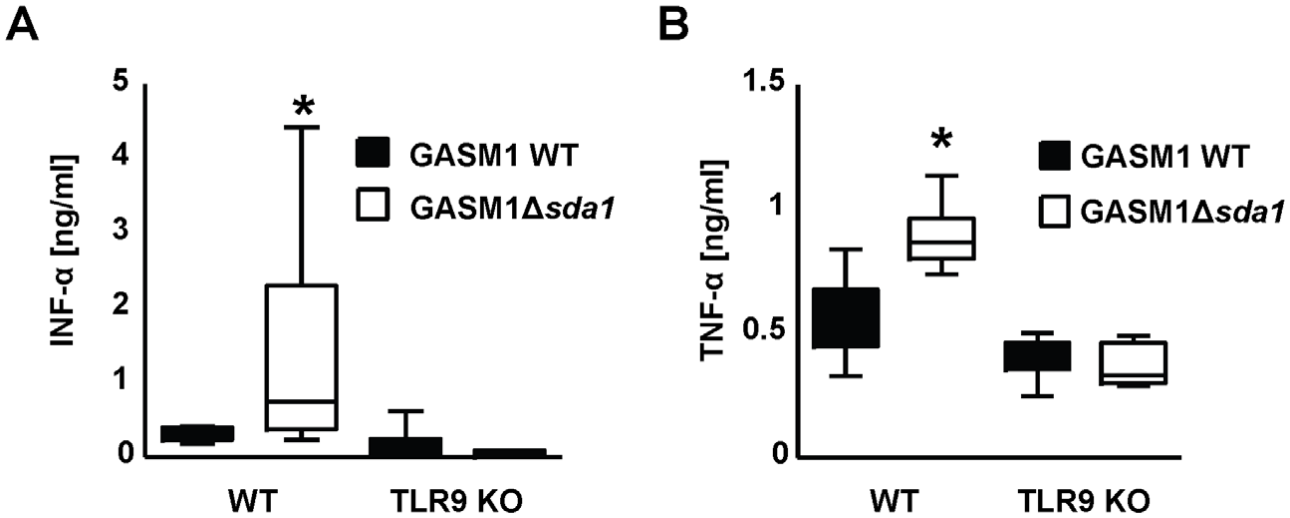
GAS DNase Sda1 diminishes local secretion of IFN-α and TNF-α in a mouse necrotizing fasciitis model. (**A** and **B**) IFN-α and TNF-α concentrations were measured by ELISA in skin homogenates of mice infected with WT GASM1 or Δ*sda1* mutant strains 4 days previously. Samples derived from WT mice infected with GASM1 WT contained significantly lower levels of IFN-α and TNF-α compared to the samples derived from WT mice infected with GASM1 Δ*sda1*. Low levels of IFN-α and TNF-α were detected in TLR-9 mice irrespective of the presence of Sda1. Data are displayed as box blots with n = 5 for the group of TLR9 mice injected with GAS Δ*sda1* and n = 6 for the other groups. Box and whiskers plot, box containing 50% with median, whiskers 2.5–97.5%. The data shown are pooled from two independent experiments. In (A) ANOVA was significant at *P<0.05, with the group of WT mice infected with GASM1 Δ*sda1* having stronger IFN-α induction (Bonferroni comparison). In (B) a factorial analysis revealed that both the mouse (*P*<0.01) and bacteria strain (*P*<0.02) as significant factors with significant interaction (*P*<0.01).

To provide *in vivo* corroboration of the *ex vivo* experiments carried out using BMDMs, we examined IFN-α and TNF-α levels in skin homogenates of mice infected subcutaneously with GAS. Despite the important contribution of Sda1 to GAS proliferation and necrotic ulcer development in this model [6], [7], WT mice infected with the GAS M1Δ*sda1* mutant showed higher levels of IFN-α and TNF-α in the skin samples than mice infected with the WT parent GAS expressing Sda1 (**Fig. 6A, B**). Parallel experiments performed in TLR9-deficient mice showed much lower cytokine levels in the infected skin compared to WT mice, again underlining the importance of TLR9 in mediating cytokine responses. However, in contrast to the WT mice, the presence or absence of Sda1 did not affect the level of cytokines produced in response to GAS in the TLR9-deficient mice (**Fig. 6A, B**). We had shown previously [14] that more bacteria are found in the skin of TLR9-deficient mice compared to WT mice, and that more surviving GASM1 WT bacteria compared to GASM1Δ*sda1* are present following experimental challenge of WT mice [6]. We also found more WT than Δ*Sda1* mutant bacteria present following injection into TLR9- deficient mice (**Fig. S8**). The observation that the TLR9-deficient mice injected with the GASM1Δ*sda1* mutant demonstrated similar bacterial counts compared to WT mice could be due to a large initial influx of neutrophils efficiently clearing the DNase-deficient mutant strain within extracellular traps. The increased cytokine levels detected in WT mice injected with GASM1Δ*sda1* mutant compared to WT bacteria are not explained by differences in bacterial counts, nor do bacterial levels account for increased cytokine levels in WT mice compared to TLR9- deficient mice. In sum, we documented that increased tissue expression of IFN-α and TNF-α in the mouse necrotizing skin infection model occurred in both a DNase- and TLR9-dependent manner.

In contrast to our *ex vivo* data and tissue culture experiments carried out by others [25], IFN-α and TNF-α secretion in our *in vivo* experiments was strictly TLR9-dependent. This finding suggests that TLR9 recognition of GAS is of true physiological relevance, and the described TLR9-independent pathways elicited *ex vivo* may be of diminished importance in the *in vivo* setting. Our experiments emphasize the critical nature of innate immune recognition at tissue foci of infection. It is important to note that while much evidence exists that IFN-α is beneficial to innate immune cells in combating bacterial infection [18], if IFN-α is produced systemically at high levels or in an uncontrolled fashion and deleterious effects on antibacterial clearance may be observed [32].

In our work we have focused on the cytokine responses and killing activities of isolated macrophages vs. GAS and the expression of cytokines at the site of GAS subcutaneous infection. We hypothesize that GAS Sda1 contributes to disease in multiple ways, including interfering with TLR9 recognition (blunting the initial innate immune response) and degrading extracellular traps (promoting phagocyte evasion). Together, these Sda1-dependent virulence phenotypes increase the risk of bacterial proliferation to produce severe necrotizing infections, septicemia or toxic shock syndrome, where ultimately high cytokine levels develop in response to the uncontrolled infection [33]. Additionally, variation in cytokine responses to GAS superantigens influences the severity of streptococcal toxic shock syndrome [34].

To summarize, we here describe a novel mechanism by which a bacterial pathogen can directly elude TLR9 recognition. The GAS virulence factor DNase Sda1 leads to decreased production of the proinflammatory cytokines IFN-α and TNF-α and to decreased killing efficiency of macrophages, which are key contributors for innate immunity to GAS infection [29]. DNase production is now recognized to be a virulence factor of a number of bacterial pathogens including *Streptococcus pneumoniae* [35], *Staphylococcus aureus* [36] and *Pseudomonas aeruginosa* [37] and it could be fruitful to determine whether evasion of TLR9-based detection complements NET degradation in the infectious pathogenesis of these species. Previously, inhibition of Sda1 activity by G-actin boosted neutrophil extracellular killing of the WT GAS bacteria and reduced lesion size in the necrotizing skin infection model, providing proof-of-principle that this DNase can represent a pharmacological target for virulence factor neutralization [6]. Our current data, demonstrating that loss of Sda1 enhances both TLR9-mediated innate immune responses and macrophage bacterial killing, provides additional rationale toward such a therapeutic strategy.

## Materials and Methods

### Mice and bacterial strains

C57BL/6 wild-type (WT) and C57BL/6 TLR9-deficient mice were bred and handled in strict accordance with the recommendations in the Guide for the Care and Use of Laboratory Animals of the National Institutes of Health. The protocol was approved by the Institutional Animal Care and Use Committee of the University of California, San Diego (Animal Welfare Assurance Number: A3033-01). All efforts were made to minimize suffering of animals employed in this study.

C57BL/6 TLR9-deficient mice were originally developed by Dr. Shizuo Akira (Osaka University, Japan); WT mice were purchased from Charles River Laboratories. The GAS strain 5448, a well-characterized M1T1 clinical isolate from a patient with necrotizing fasciitis and toxic shock syndrome [38] and the GAS M49 strain NZ131 [39] were used. In addition, to analyze gain and loss of function of Sda1, GAS strains expressing Sda1 (GASM1T1 WT, GASM1T1 Δ*sda1*pDc*sda1* and GASM49 pDc*sda1*) and lacking Sda1 (GASM1T1 Δ*sda1* and GASM49 WT pDc*erm*) were used in the assays as described previously [6], [40] Growth curves showed that all strains used in the experiments grew identically. GAS strains were propagated in Todd-Hewitt broth (THB) (Difco, BD Diagnostics) or Todd-Hewitt agar plates. For use in macrophages and mouse challenge studies, bacteria were grown to logarithmic phase in THB (OD_600_ = 0.4 corresponding to ∼2×10^8^ cfu/ml), pelleted, washed and resuspended in PBS or tissue culture media at the desired concentration.

### GAS and human genomic DNA preparation

GAS genomic DNA was prepared using Bactozol kit (Molecular Research Center Inc.) with minor modifications. GAS strains were incubated overnight in THB medium, 2 ml of the overnight culture pelleted and resuspended in Bactozol buffer+50 U mutanolysin and 10 U Proteinase K, then incubated at 45°C for 90 min. Bactozol enzyme was added and the preparation incubated for 60 min at 45°C. From this step on, the standard Bactozol kit protocol was followed. Human genomic DNA was prepared from buffy coats of healthy volunteers from the blood donation center in Zurich, Switzerland. Cells were pelleted by centrifugation at 1000 g for 10 min. Genomic DNA was then extracted using blood and tissue DNA easy kit (Qiagen) following the manufacturer's protocol. Remaining RNA was digested by adding RNase during DNA purification. Purity of the isolated DNA, bacterial or human, was confirmed by lack of any cytokine stimulation after digesting the DNA with DNase I (Roche) (Fig. S5). Genomic DNA integrity was confirmed by agarose gel.

### Sda1 production and purification

The *sda1* gene was amplified from GAS M1T1 genomic DNA using primers forward 5′-TCGAGCTCTCTAAACATTGGAGACATCTAATTATTCACTCTG-3′ and reverse 5′-TGGTCGACTTATTCTATATTTTCTTGAGTTGAATGATG-3′. The PCR product was subcloned into vector pQE30 and the newly created plasmid transformed into the *E. coli* strain M15-pREP4 (Qiagen) for protein production. Bacteria were grown to OD_600_ = 0.5 at 30°C in the presence of 100 µg/ml ampicillin and 25 µg/ml kanamycin and the expression of Sda1 induced for 1 h via addition of 1 M IPTG. Bacteria were then harvested, resuspended in 30 ml lysis buffer (50 mM NaH_2_PO_4_, 300 mM NaCl, 20 mM imidazole pH 8) and lysed by sonication at full power (20 cycles of 15 seconds each). Cell debris were spun down at 12,000 g for 30 min, the supernatant filtered through a 0.45 µm PVDF filter, then run on a HiTrap nickel bead column (GE Healthcare) at a 1 ml/min speed. The column was then washed with 10 volumes lysis buffer and with 10 volumes wash buffer (50 mM NaH_2_PO_4_, 300 mM NaCl, 50 mM imidazole pH 8). Sda1 was eluted with 5 ml elution buffer (50 mM NaH_2_PO_4_, 300 mM NaCl, 250 mM imidazole pH 8), dialysed against storage buffer (50% glycerol in PBS) and stored at −20°C.

### Zymogram

The quality and activity of the purified recombinant Sda1 were checked by performing a zymogram. Briefly, 1 µg of Sda1 and 1 µg of DNaseI, used as a control for activity, were loaded on a 12% polyacrylamide gel containing 10 µg/ml of calf thymus DNA. The gel was washed 2 times in ddH_2_O and then incubated overnight in 50 mM TRIS·HCl pH 7.4. The gel was then incubated (35 hours, 37°C) in Sda1 reaction buffer (50 mM TRIS·HCl pH 7.4, 5 mM CaCl_2_) containing 1 µg/ml EtBr. DNA degradation showed on gel as a dark band and was taken as a proof of nuclease activity.

### Pretreatment of genomic bacterial DNA with nucleases

Genomic DNA (2.5 µg) was incubated with DNase I from bovine pancreas (Roche) at 37°C for 6 hours. Furthermore, 2.5 µg of genomic DNA were incubated with purified recombinant Sda1 at 37°C for 0 to 6 hours. After 1, 2, 4 and 6 hours, 1 M EDTA was added to stop the reaction and the samples were loaded on an agarose gel or added to the BMDMs. DNA alone and DNase buffer without adding recombinant Sda1 served as controls. Degradation of genomic DNA was confirmed by agarose gel electrophoresis.

### GAS DNA degradation assay by bacteria supernatants

The GAS DNase Sda1 activity was tested as previously described with minor modifications [6]. 5 µl of a 1∶100 dilution of filtered supernatants taken at OD_600_ 0.4 were mixed with 3 µl reaction buffer (50 mM Tris-HCl, 5 mM CaCl), 20 µl of water and 2 µl of GAS genomic DNA (125 ng/µl) and incubated for five minutes at 37°C. The reaction was stopped by addition of 1 M EDTA. The DNA was run on 1% agarose gel for visualisation.

### Mice bone marrow derived macrophages isolation

Murine bone marrow-derived macrophages (BMDMs) were isolated as previously described [41] with some modifications. Bone marrow cells were collected from mice legs and cultured for 7 days in Dulbecco's modified Eagle's medium (high glucose) supplemented with 30% L-929 cell conditioned medium. The adherent cells (BMDM) were then collected, split to assay settings, and cultured in Dulbecco's modified Eagle's medium (high glucose) until being used for experiments on day 10.

### Macrophage challenge with genomic DNA or bacteria

BMDM (5×10^5^) were seeded into each well of a 48 well plate in 500 µl medium. On day 10, 2 h before the inoculation of genomic DNA or bacteria, BMDM were washed twice with PBS, and 200 µl DMEM+10% FBS (70°C heat inactivated) were added to each well. GAS strains and genomic DNA, prepared as described above, were inoculated into wells at a multiplicity of infection (MOI) of 1 and 5 µg/ml respectively. In addition 80 µl of the DNA samples obtained after digestion with rSda1 for 0, 1, 2, 4 and 6 hours were used. Media without addition of DNA or rSda1 as well as media containing rSda1 alone served as controls. Plates inoculated with bacteria were centrifuged at 800 g for 10 min, incubated at 37°C in a CO_2_ incubator for 2 h, and penicillin G and gentamicin added to each well to a concentration of 10 and 100 µg/ml, respectively. As TLR9 specific agonist and antagonist, 5 µg/ml CpG-ODN 2395 (Microsynth, 5′-tcg tcg ttt tcg gcg gcg ccg-3′ with phosphorothioate on all bases) and G-ODN (Microsynth, 5′- ctc cta ttg ggg gtt tcc tat -3′ with phosphorothioate on all bases) were used. Challenged macrophages were incubated at 37°C with 5% CO_2_ for 12 h. The plates were centrifuged at 800 g for 10 min before the supernatants were taken and stored in −80°C until they were used in ELISA assays.

### Cytokine measurements

IFN-α and TNF-α ELISA: Levels of IFN-α in culture supernatants were analyzed by a standard sandwich ELISA using a monoclonal mouse IFN-α capture antibody (Hycult biotech) and polyclonal rabbit IFN-α antibody (PBL interferon source) together with a goat anti-rabbit antibody conjugated with HRP (Invitrogen). A serial dilution of recombinant mouse IFN-α (PBL interferon source) was used to calculate the absolute concentration in the supernatants. Levels of TNF-α were measured using mouse TNF-α ELISA kit (R&D) following the manufacturer's protocol.

Interferon type 1 cell luciferase assay: Luciferase cell reporter assay of IFN-1 was carried out using the LL171 luciferase reporter cell line as described [42].

### Macrophage killing assays and oxidative burst expression

Macrophages were harvested and seeded in 48 well plates as described above. Two hours before adding bacteria, macrophages were washed twice with PBS and 400 µl of DMEM+2% FBS were added to each well. Logarithmic phase bacteria were added to the wells at final MOI of 1 and plates were centrifuged for 5 minutes at 1500 rpm. For total killing, the plate was incubated for 4 hours. For intracellular killing assays, 100 µg/ml penicillin G and 100 µg/ml gentamicin were added to the wells and the plate was incubated for another 2 h at 37°C in 5% CO_2_ before macrophages were detached with trypsin and lysed with 0.025% Triton-X100. Serial dilutions of the lysates were plated on THA for enumeration of surviving bacterial colony forming units (cfu). Reactive oxygen species were measured following the protocol described before [14].

### Murine infection model

An established murine model of necrotizing skin infection was used [43]. Briefly, logarithmic phase GAS were resuspended in PBS, mixed 1∶1 with sterile Cytodex beads (Sigma) and an inoculum of 5×10^7^ cfu of GAS was injected subcutaneously into one flank of 10–12 week old WT or TLR9-deficient mice. At day four the mice were euthanized and skin from the lesion was collected, homogenized and IFN-α and TNF-α measured by ELISA and bacterial counts assessed after serial dilutions and plating on THA plates.

### Statistics

Data were analysed and edited using the SPSS (SPSS 11.5 Inc., Chicago, Illinois, USA), the NCSS (Kaysville, Utah, USA) and Graphpad prism 5 software (Graphpad Software Inc, La Jolla, California, USA) packages. Two-sample two-tailed homoscedastic t-tests were used to calculate the indicated p-values except for the animal studies (Fig. 6) for which analysis of variance (ANOVA) followed by Bonferroni comparison and a factorial analysis (2-way ANOVA) were used to calculate indicated p-values.

## Supporting Information

Figure S1 **No degradation of RNA was observed by recombinant GAS DNase Sda1.** RNA was isolated from GAS and co-incubated with either the DNase buffer alone or with 365 ng of the recombinant Sda1 in DNase buffer for 10 minutes at 37°C. Visualisation followed by 1.5% TBE agarose gel electrophoresis.
(TIF)

Figure S2 **The recombinant GAS DNase Sda1 does not induce IFN-α and TNF-α secretion.** rSda1 was co-incubated with murine macrophages and the cytokine response measured after 12 hours.
(TIF)

Figure S3 **The GAS DNase Sda1 interferes with TLR9 activation.** Stimulation for 12 hours of BMDMs with GAS strains expressing Sda1 (GASM1 WT and GASM1 Δ*sda1* pD*csda1*) resulted in significantly less IFN-α and TNF-α secretion compared to matching strains lacking Sda1 (GASM1 Δ*sda1*). Data were pooled from 3 experiments done in triplicates and presented as mean ± SEM.
(TIFF)

Figure S4 **The GAS DNase Sda1 does not affect extracellular killing in macrophages.**
(TIF)

Figure S5 **GAS DNA digested with DNaseI does not induce IFN-α and TNF-α secretion.** In order to test for purity of our isolated bacterial DNA we co-incubated the bacterial DNA with and without DNaseI and stimulated BMDMs for 12 hours. Addition of DNaseI resulted in similarly low IFN-α and TNF-α secretion as observed for the controls.
(TIF)

Figure S6 **BMDMs viability assays.** Logarithmic phase bacteria were added to BMDM at MOI 1. Recombinant streptolysin O (rSLO) was used as a positive control at a final concentration of 16 µg/ml. After 4 (**A**) and 12 hours (**B**) BMDMs were analysed using the mammalian cell live/dead staining kit or the MTT assay at 4 (**C**) and 12 hours (**D**) respectively. Data were pooled from 3 experiments done in triplicates and presented as mean ± SEM.
(TIF)

Figure S7 **Addition of neutralizing antibodies against INF-α increases bacterial survival of GAS.** In order to test if blocking IFN-α and TNF-α can prevent phagocytic killing mediated by GASΔ*sda1* we repeated the BMDM killing assays using with WT BMDM challenged with GASWT M1 and GASΔ*sda1* bacteria (MOI 1) after having pre-incubated the BMDM for 2 h with either the neutralizing antibodies against TNF-α or IFN-α or their respective controls. Data were pooled from 3 experiments done in triplicates and presented as mean ± SEM. ** *P*<0.01.
(TIF)

Figure S8 **TLR9 is important for controlling GAS infection** ***in vivo*** **.** WT and TLR9-deficient mice were injected subcutaneously with equivalent inocula of GASWT M1 and GASΔ*sda1* and after 4 days bacteria were enumerated in the skin. N = 5 for the group of TLR9 mice injected with GAS Δ*sda1* and n = 6 for the other groups. Data shown were pooled from two independent experiments and presented as mean ± SEM.
(TIF)

Text S1 **Contains supplementary methods of the supplemental data.**
(DOC)
