# Supplementary figures and images for: DNase Sda1 Allows Invasive M1T1 Group A Streptococcus to Prevent TLR9-Dependent Recognition
Source: PLoS Pathog. 2012 Jun 14;8(6):e1002736. doi: 10.1371/journal.ppat.1002736 (PMC3375267; doi:10.1371/journal.ppat.1002736)

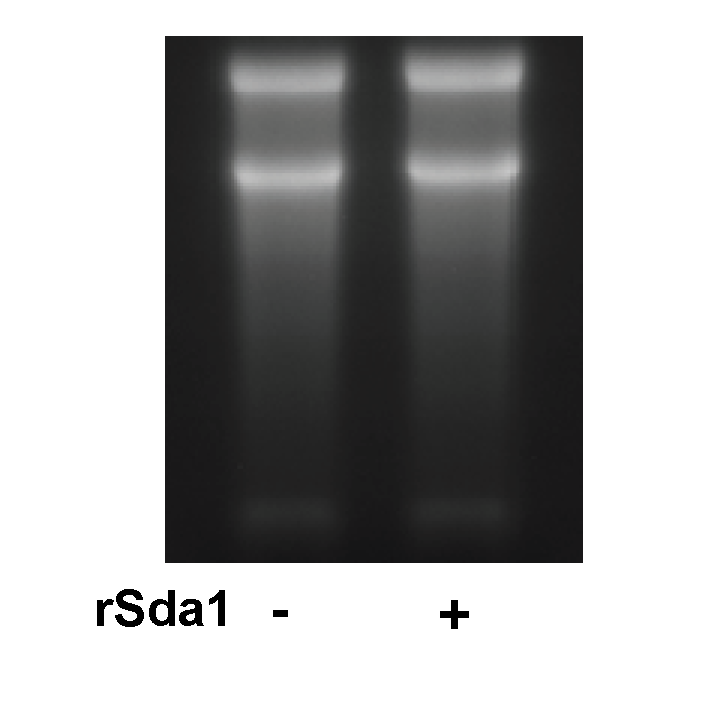

Supplement: Figure S1 — No degradation of RNA was observed by recombinant GAS DNase Sda1. RNA was isolated from GAS and co-incubated with either the DNase buffer alone or with 365 ng of the recombinant Sda1 in DNase buffer for 10 minutes at 37°C. Visualisation followed by 1.5% TBE agarose gel electrophoresis. (TIF) [file ppat.1002736.s001.tif]

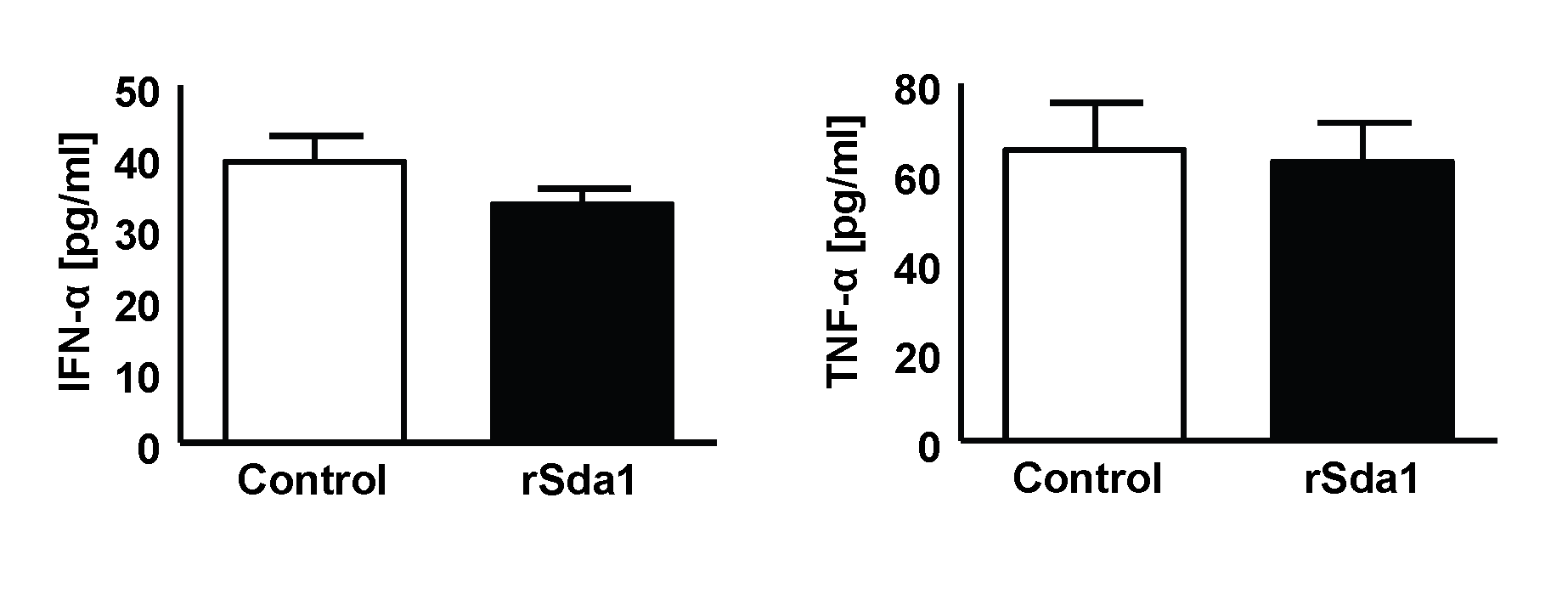

Supplement: Figure S2 — The recombinant GAS DNase Sda1 does not induce IFN-α and TNF-α secretion. rSda1 was co-incubated with murine macrophages and the cytokine response measured after 12 hours. (TIF) [file ppat.1002736.s002.tif]

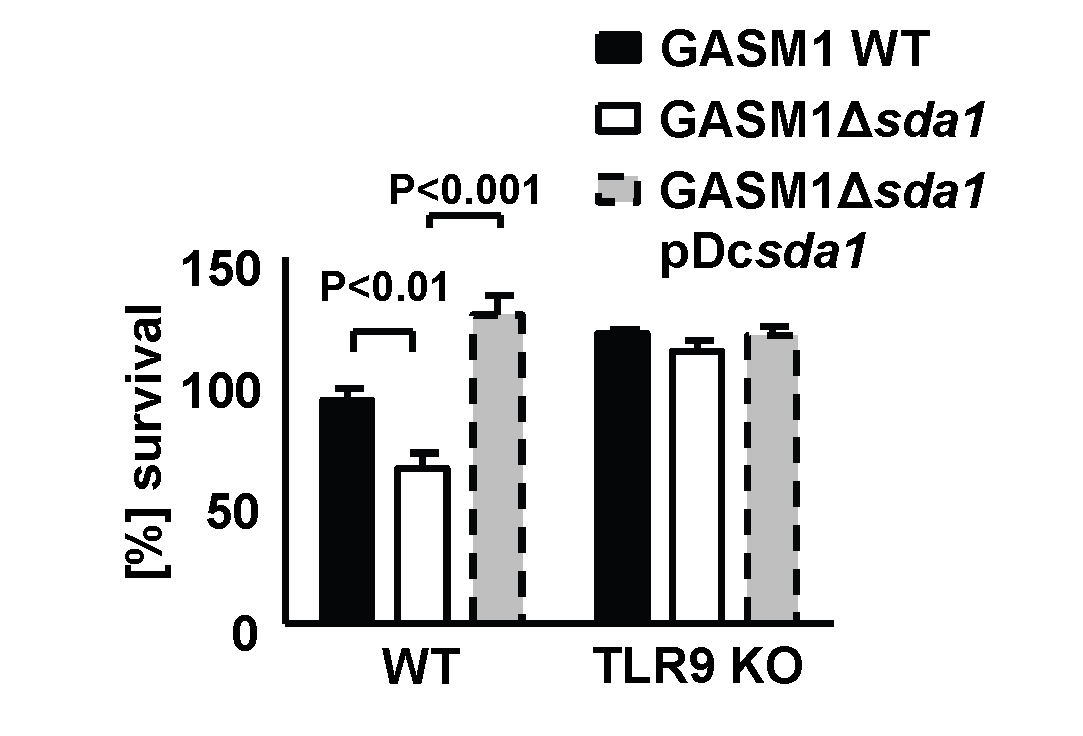

Supplement: Figure S3 — The GAS DNase Sda1 interferes with TLR9 activation. Stimulation for 12 hours of BMDMs with GAS strains expressing Sda1 (GASM1 WT and GASM1 Δsda1 pDcsda1) resulted in significantly less IFN-α and TNF-α secretion compared to matching strains lacking Sda1 (GASM1 Δsda1). Data were pooled from 3 experiments done in triplicates and presented as mean ± SEM. (TIFF) [file ppat.1002736.s003.tif]

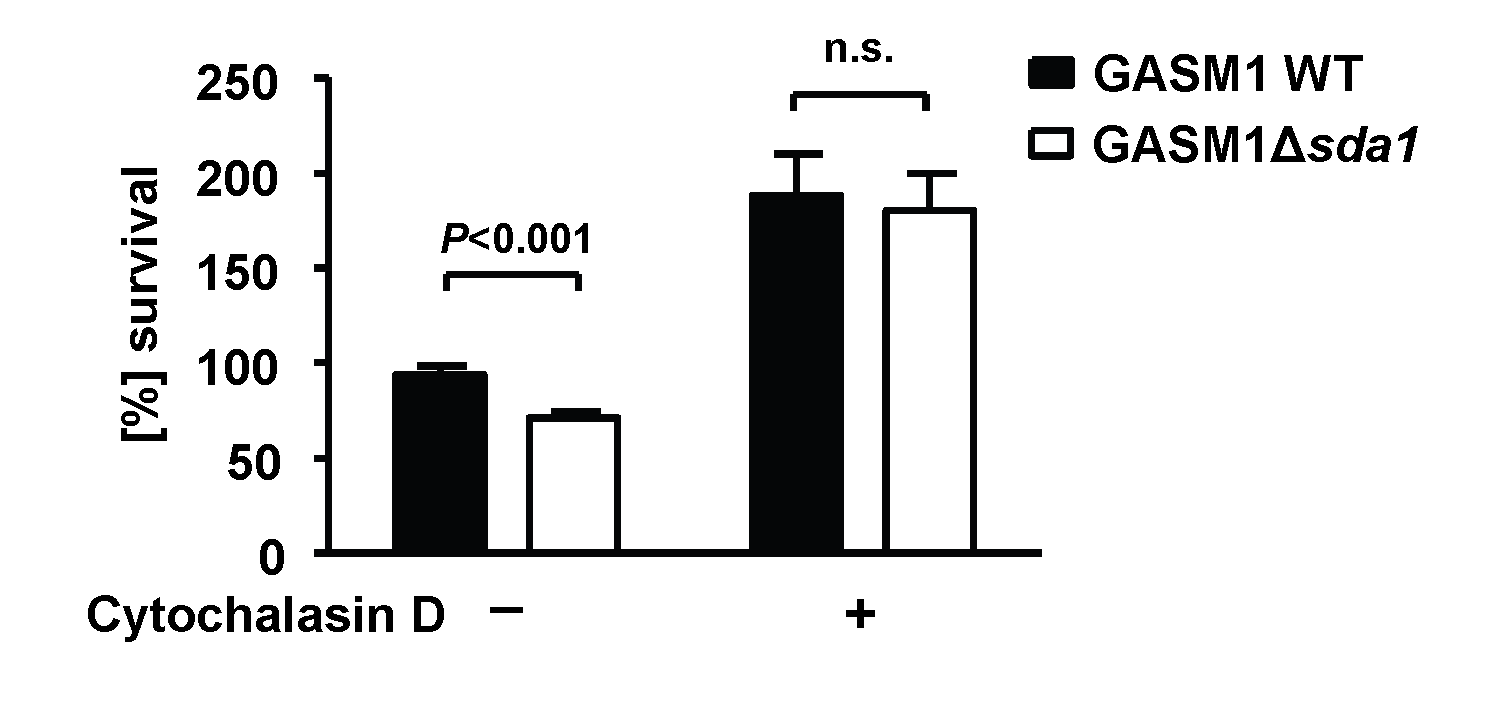

Supplement: Figure S4 — The GAS DNase Sda1 does not affect extracellular killing in macrophages. (TIF) [file ppat.1002736.s004.tif]

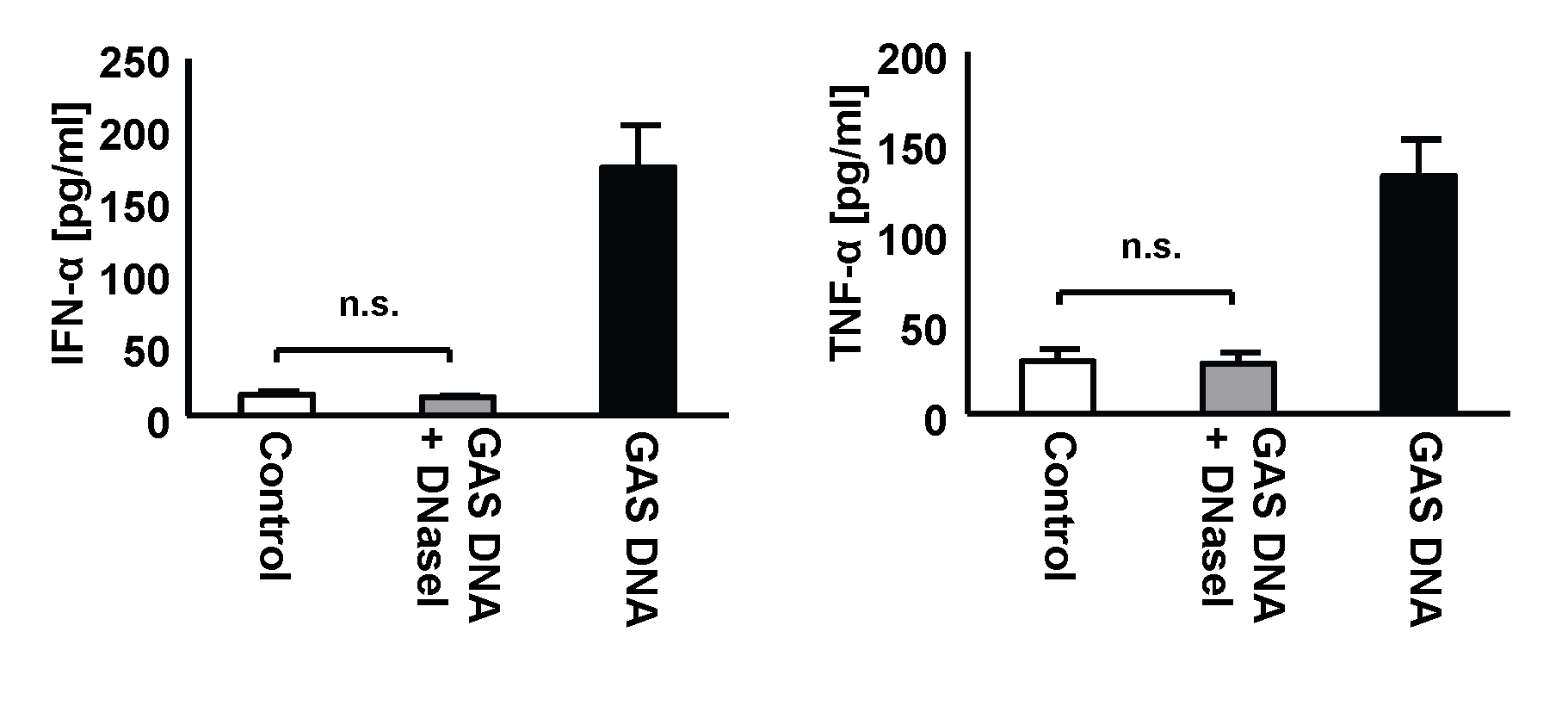

Supplement: Figure S5 — GAS DNA digested with DNaseI does not induce IFN-α and TNF-α secretion. In order to test for purity of our isolated bacterial DNA we co-incubated the bacterial DNA with and without DNaseI and stimulated BMDMs for 12 hours. Addition of DNaseI resulted in similarly low IFN-α and TNF-α secretion as observed for the controls. (TIF) [file ppat.1002736.s005.tif]

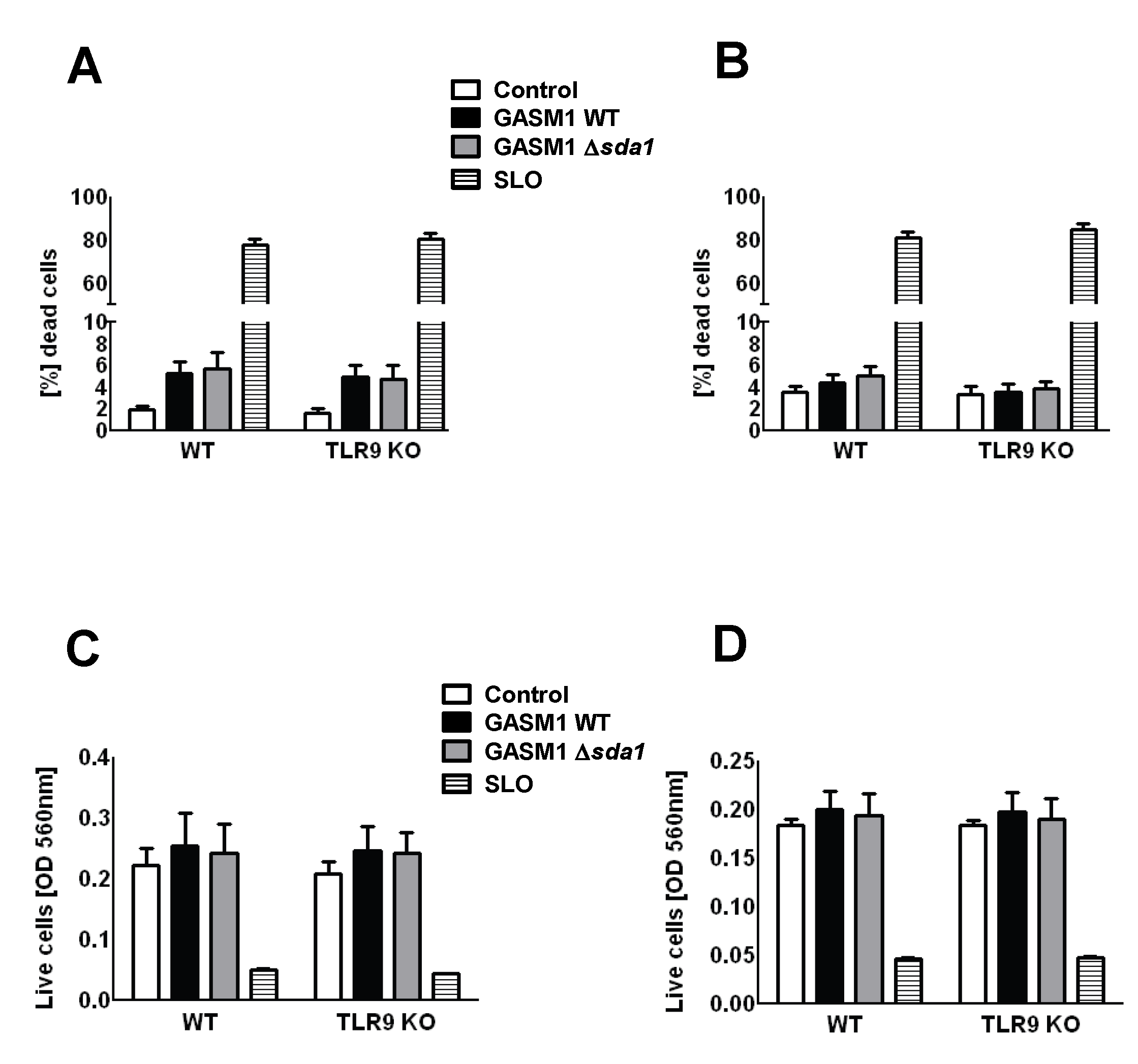

Supplement: Figure S6 — BMDMs viability assays. Logarithmic phase bacteria were added to BMDM at MOI 1. Recombinant streptolysin O (rSLO) was used as a positive control at a final concentration of 16 µg/ml. After 4 (A) and 12 hours (B) BMDMs were analysed using the mammalian cell live/dead staining kit or the MTT assay at 4 (C) and 12 hours (D) respectively. Data were pooled from 3 experiments done in triplicates and presented as mean ± SEM. (TIF) [file ppat.1002736.s006.tif]

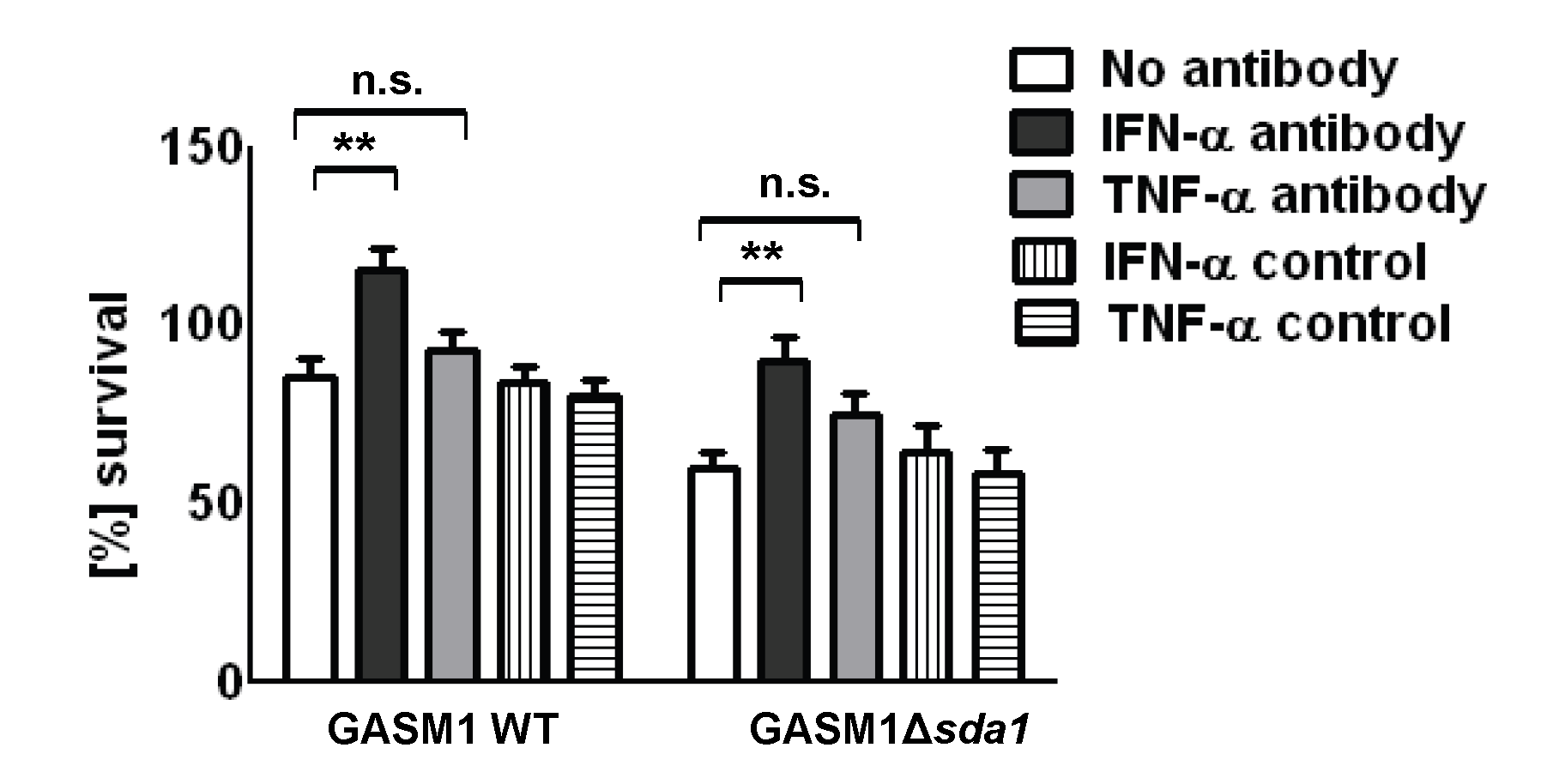

Supplement: Figure S7 — Addition of neutralizing antibodies against INF-α increases bacterial survival of GAS. In order to test if blocking IFN-α and TNF-α can prevent phagocytic killing mediated by GASΔsda1 we repeated the BMDM killing assays using with WT BMDM challenged with GASWT M1 and GASΔsda1 bacteria (MOI 1) after having pre-incubated the BMDM for 2 h with either the neutralizing antibodies against TNF-α or IFN-α or their respective controls. Data were pooled from 3 experiments done in triplicates and presented as mean ± SEM. ** P<0.01. (TIF) [file ppat.1002736.s007.tif]

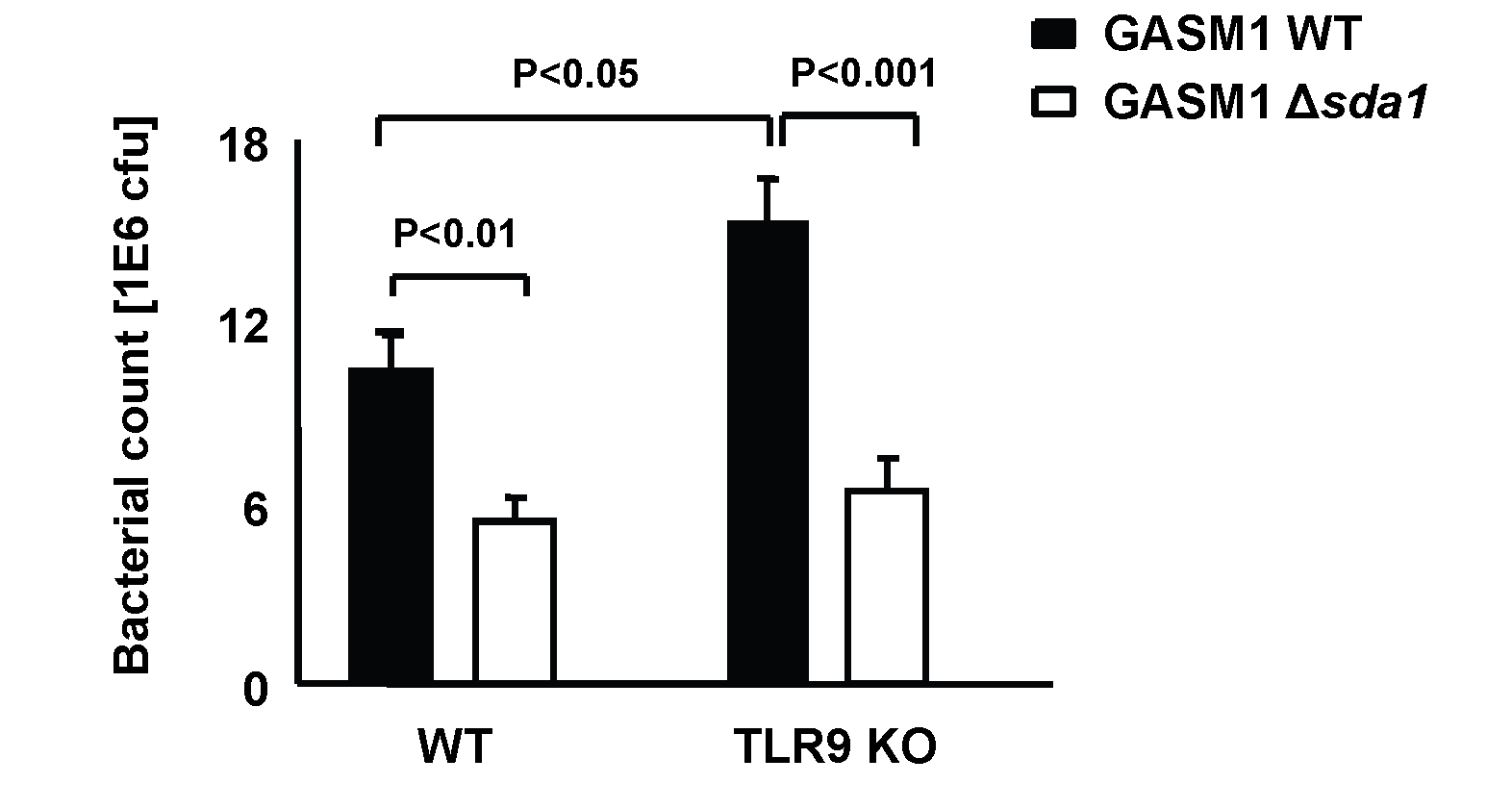

Supplement: Figure S8 — TLR9 is important for controlling GAS infection in vivo . WT and TLR9-deficient mice were injected subcutaneously with equivalent inocula of GASWT M1 and GASΔsda1 and after 4 days bacteria were enumerated in the skin. N = 5 for the group of TLR9 mice injected with GAS Δsda1 and n = 6 for the other groups. Data shown were pooled from two independent experiments and presented as mean ± SEM. (TIF) [file ppat.1002736.s008.tif]
