# Supplementary material for: DNase Sda1 Allows Invasive M1T1 Group A Streptococcus to Prevent TLR9-Dependent Recognition
Source: PLoS Pathog. 2012 Jun 14;8(6):e1002736. doi: 10.1371/journal.ppat.1002736 (PMC3375267; doi:10.1371/journal.ppat.1002736)
Supplement: Text S1 — Contains supplementary methods of the supplemental data. (DOC) [file ppat.1002736.s009.doc]

**Supplemental Materials and Methods**

**Pretreatment of RNA with recombinant Sda1**

RNA was prepared from GAS using the Qiagen RNAeasy kit following the manufacturer’s instructions. Five μg of GAS RNA were then incubated at 37°C for 10 minutes with either the DNase buffer alone or with 365 ng of the recombinant sda1 in DNase buffer . Visualisation followed by 1.5% TBE agarose gel electrophoresis.

**Macrophage extracellular killing assays**

For extracellular killing the BMDMs were incubated with 10 μg/ml cytochalasin D (Sigma) for 1 hour. After this incubation period, the total killing assay protocol was followed. Serial dilutions of the lysates were plated on THA for enumeration of surviving bacterial colony forming units (cfu). Reactive oxygen species were measured as described before . BMDM killing assays were repeated with WT BMDM pretreated with the neutralizing antibodies against TNF-α or IFN-α. Ten μg/ml anti murine TNF-α antibody (clone XT3.11, BioXcell) or 1 μg/ml anti murine IFN-α antibody (PBL interferon source) were added to the BMDMs 2h before the GAS killing assay was performed as described in the methods section. Rat anti-human monoclonal antibody (Hycult biotech) for TNF-α and polyclonal rabbit IgG control (R&D) for IFN-α served as controls.

**BMDMs viability assays**

Macrophages were harvested as described above. Logarithmic phase bacteria were added to the wells at final MOI of 1 and plates were centrifuged for 5 minutes at 1500 rpm. The same time points as for the assays above were used (i.e. 4 hours as for the bacterial killing assay and 12 hours as for the cytokine stimulation assays). Recombinant streptolysin O (rSLO) was used as a positive control at a final concentration of 16 μg/ml. Since rSLO lyses the cells completely after 12 hours rSLO was used only for 4hours for both time points.

**Live/dead mammalian cell staining**: BMDMs were seeded at 1 x 10^5^ cells/well into black 96 well plates with glass bottoms and challenged with bacteria as described above. After 4 and 12 hours, respectively, the BMDMs were washed with PBS 3 times and the mammalian cell live/dead staining (Invitrogen) was performed following the manufacturer’s instructions. The BMDMs were visualised with the fluorescent IX71 microscope. At least 9 pictures per condition were randomly taken for analysis. For data analysis, the total number of BMDMs per picture was assessed as well as dead BMDMs and the ratio of dead cells/total cells (%) was calculated.

**MTT assay**: BMDMs were split into 96 well plates at 1 x 10^5^ cells / well and challenged with bacteria as described above. After 4 and 12 hours respectively, the wells were washed 3 times with HBSS with Ca^2+^ and Mg^2+^ before 120 μl DMEM+10% FCS and 30μl of 5 mg/ml MTT solution (Sigma) were added to each wells. The plate was then incubated at 37^o^C in 5% CO_2_. The supernatant was removed and 100µl/well 0.04 M HCl in isopropanol was added to the wells and was incubated in RT for 15 minutes and the OD_560_ nm measured.

**REFERENCES**

1. Zinkernagel AS, Hruz P, Uchiyama S, von Kockritz-Blickwede M, Schuepbach RA, et al. (2011) Importance of Toll-Like Receptor 9 in Host Defense against M1T1 Group A Streptococcus Infections. J Innate Immun.

2. Timmer AM, Timmer JC, Pence MA, Hsu LC, Ghochani M, et al. (2009) Streptolysin O promotes group A Streptococcus immune evasion by accelerated macrophage apoptosis. J Biol Chem 284: 862-871.
